# HIV-1 Vpu utilizes both cullin-RING ligase (CRL) dependent and independent mechanisms to downmodulate host proteins

**DOI:** 10.1186/s12977-015-0192-2

**Published:** 2015-07-28

**Authors:** Peter W Ramirez, Ana Beatriz DePaula-Silva, Matt Szaniawski, Edward Barker, Alberto Bosque, Vicente Planelles

**Affiliations:** Division of Microbiology and Immunology, Department of Pathology, University of Utah School of Medicine, Salt Lake City, UT 84112 USA; Department of Immunology and Microbiology, Rush University Medical Center, Chicago, IL 60612 USA

**Keywords:** CRL, Vpu, MLN4924, Nef, CD4, BST-2, NTB-A, CCR7

## Abstract

**Background:**

Hijacking of the cullin-RING E3 ubiquitin ligase (CRL) machinery is a common mechanism employed by diverse groups of viruses for the efficient counteraction and degradation of host proteins. In particular, HIV-1 Vpu usurps the SCF^β-TrCP^ E3 ubiquitin ligase complex to mark CD4 for degradation by the 26S proteasome. Vpu also interacts with and downmodulates a number of other host proteins, including the restriction factor BST-2. However, whether Vpu primarily relies on a cullin-dependent or -independent mechanism to antagonize its cellular targets has not been fully elucidated.

**Results:**

We utilized a sulphamate AMP analog, MLN4924, to effectively block the activation of CRLs within infected primary CD4^+^ T cells. MLN4924 treatment, in a dose dependent manner, efficiently relieved surface downmodulation and degradation of CD4 by NL4-3 Vpu. MLN4924 inhibition was highly specific, as this inhibitor had no effect on Nef’s ability to downregulate CD4, which is accomplished by a CRL-independent mechanism. In contrast, NL4-3 Vpu’s capacity to downregulate BST-2, NTB-A and CCR7 was not inhibited by the drug. Vpu’s from both a transmitted founder (T/F) and chronic carrier (CC) virus preserved the ability to downregulate BST-2 in the presence of MLN4924. Finally, depletion of cellular pools of cullin 1 attenuated Vpu’s ability to decrease CD4 but not BST-2 surface levels.

**Conclusions:**

We conclude that Vpu employs both CRL-dependent and CRL-independent modes of action against host proteins. Notably, we also establish that Vpu-mediated reduction of BST-2 from the cell surface is independent of β-TrCP and the CRL- machinery and this function is conserved by Vpu’s from primary isolates. Therefore, potential therapies aimed at antagonizing the activities of Vpu may need to address these distinct mechanisms of action in order to achieve a maximal effect.

## Background

Cullin-RING ligases (CRLs) constitute an important group of ubiquitin ligases and play a prominent role in the efficient regulation of protein turnover and homeostasis [1]. In particular, a recurring theme among viruses from distant families is their common ability to usurp CRL complexes with the aim of evading host control mechanisms. Notably, the HIV-1 accessory protein Vif hijacks a cullin-5 containing ubiquitin ligase complex (CRL5) to target cytidine deaminases of the APOBEC3 family for proteasomal degradation [2–5]. Similarly, the HIV-2 accessory protein Vpx relies on a CRL4 complex to degrade the restriction factor SAMHD1 [6, 7].

Activation of CRLs is dependent on a process known as neddylation. This post-translational modification involves the covalent addition of the NEDD8 protein, a relative of ubiquitin, onto a lysine residue on the cullin backbone. Neddylation induces a conformational change in the CRL complex that turns the enzyme catalytically active, allowing the transfer of ubiquitin to a substrate [8]. MLN4924, a potent inhibitor of the E1 neddylation enzyme NAE (Nedd8-activating enzyme), blocks the activity of all CRLs but does not affect non-cullin ubiquitin ligases [9]. Previous studies have shown that MLN4924 can potently block Vif-mediated proteasomal degradation of APOBEC3G [10]. Furthermore, in the context of HIV-2, MLN4924 inhibited the degradation of SAMHDI induced by Vpx, phenocopying the absence of Vpx in HIV-2 and restoring efficient restriction of the virus in myeloid cells [11–13].

The HIV-1 accessory protein Vpu, in addition to counteracting the restriction factor BST-2/tetherin [14, 15] and downregulating CD4, antagonizes multiple immune system molecules. Binding of Vpu’s phospho-serine residues to the F-box protein β-ΤrCP forms an SCF^β-TrCP^ (CRL1) complex that targets CD4 for proteasomal degradation [16, 17]. With regards to BST-2, counteraction is thought to be triggered by Vpu’s acidic di-leucine motif manipulating Adaptor-Protein 1 (AP-1) to mislocalize BST-2 towards a perinuclear compartment (*trans*-Golgi network-TGN) [18, 19]. However, the requirement for β-TrCP in BST-2 antagonism by Vpu has remained controversial. For other Vpu targets, specifically NTB-A and CCR7, a cullin-independent mechanism of downregulation has been proposed [20, 21].

In this study, we asked whether pharmacological inhibition of the SCF^β-TrCP^ complex by MLN4924 would reveal whether cullin activity is important for Vpu to downmodulate its cellular targets. We hypothesized that downregulation of BST-2, CCR7 and NTB-A by Vpu would not be impacted by MLN4924 treatment. Moreover, we predicted that Vpu downregulation (and degradation) of CD4 would be relieved by MLN4924. Finally, we sought to determine whether Vpu-mediated cell surface downregulation of BST-2 is a function that can be dissociated from BST-2 degradation and that is cullin-independent.

## Results

### Pharmacological inhibition of CRL-activity disables NL4-3 Vpu’s ability to downregulate CD4, but not BST-2, CCR7 or NTB-A

**Fig. 1:**
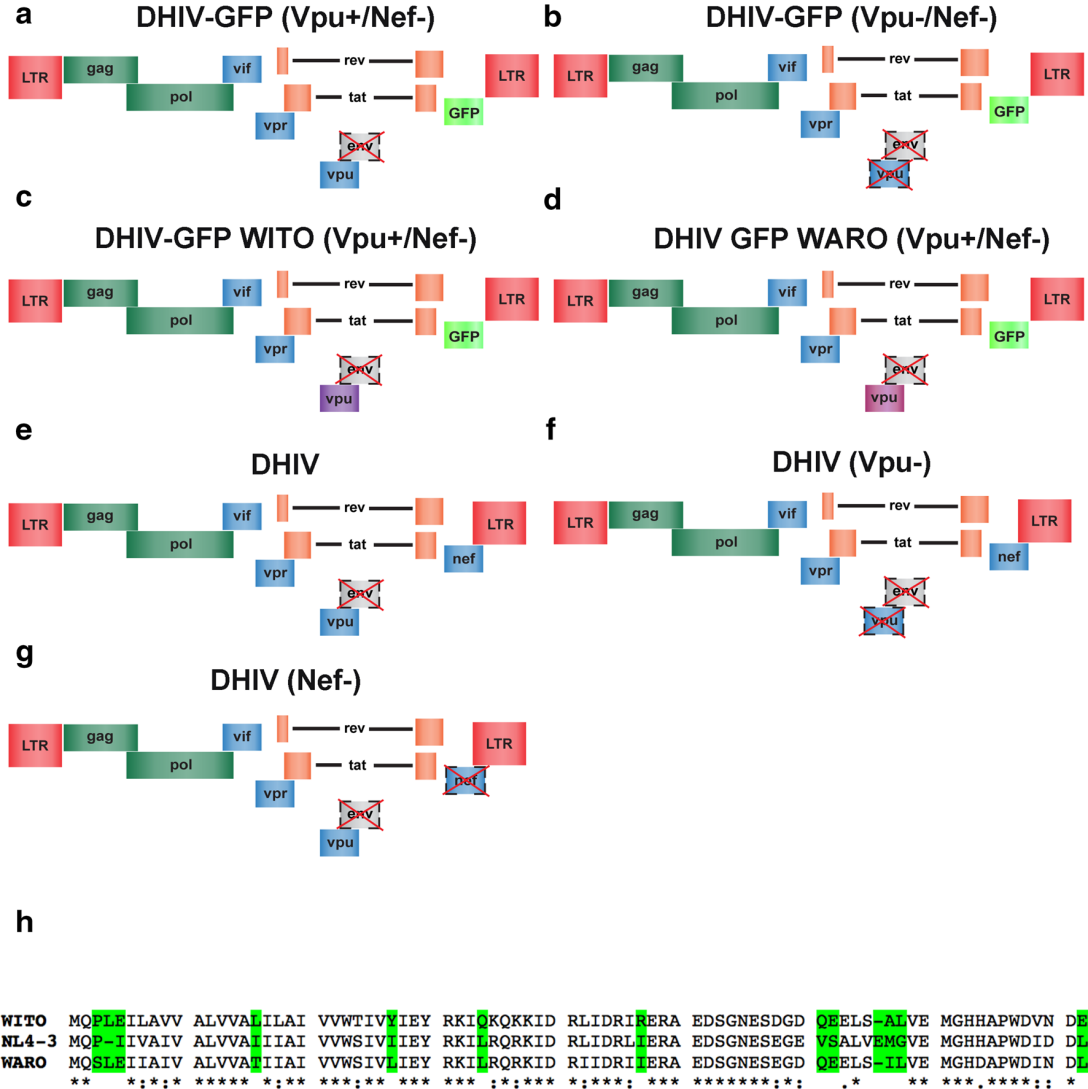
Lentiviral constructs and Vpu amino acid alignments. **a** The lentiviral vector DHIV, or “defective” HIV, was derived from the HIV-1_NL4-3_ sequence and cut between two BglII sites to efficiently delete envelope/gp120 (*gray box with dashed lines*, *red* X marks) but maintain in-frame Tat, Rev and RRE ORFs. The constructs used in this study were derived from the DHIV backbone and are as follows: *i* the GFP gene in place of Nef. *ii* Replacement of NL4-3 Vpu with a primary Vpu isolate or *iii* introduction of a frame shift mutation within Vpu and Nef. **a** DHIV-GFP (Vpu+/Nef−). **b** DHIV-GFP (Vpu−/Nef−). **c** DHIV-GFP WITO (Vpu+/Nef−). **d** DHIV-GFP WARO (Vpu+/Nef−). **e** DHIV. **f** DHIV (Vpu−). **g** DHIV (Nef−). **h** Amino acid sequence alignment of Vpu proteins from a transmitted founder (T/F;WITO) and chronic carrier (CC; WARO) compared to NL4-3 Vpu. An *asterisk* indicates fully conserved residues; *colon* represents amino acid conservation with strongly similar properties; *period* designates amino acid conservation with weakly similar properties. *Highlighted* residues mark amino acid differences between the three strains.

**Fig. 2:**
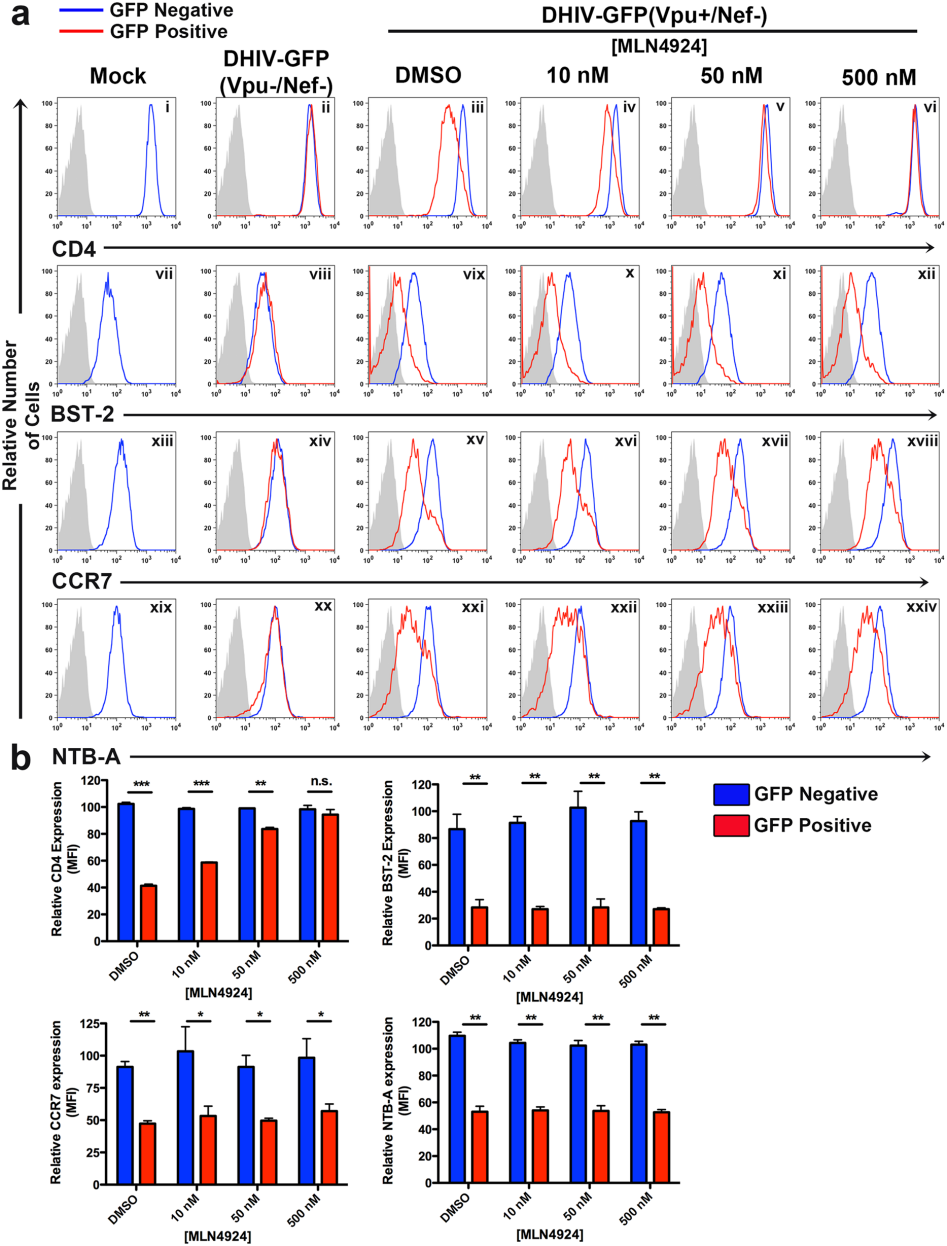
HIV-1 Vpu utilizes both cullin dependent and independent mechanisms to downregulate host proteins. **a** Primary CD4^+^ T cells were either mock infected or infected at an MOI of 1 with DHIVGFP(Vpu+Nef−) or DHIVGFP(Vpu−Nef−). 2 days post infection, either DMSO or increasing concentrations of MLN4924 were added to cell cultures. 24 h later, surface expression of CD4, BST-2, CCR7 or NTB-A was analyzed by flow cytometry. Histograms depict a comparison of GFP negative (*blue line*) and GFP positive (*red line*) cells along with an IgG isotype control (*gray* shaded histogram). Unless otherwise noted, all experiments involving primary CD4^+^ T cells are representative of three separate experiments performed in three different healthy donors. **b** Relative mean fluorescence intensity (MFI) values of surface expression of CD4, BST-2, CCR7 or NTB-A from DHIVGFP(Vpu+Nef−) infected cells (**a**). Data was normalized by setting the MFI values from uninfected (mock) cells to 100% and is depicted graphically as ±SEM. Unless otherwise noted, all experiments including statistics were performed through a pairwise Student’s t test comparing GFP positive and GFP negative cells to assess significance: *p < 0.05, **p < 0.01, ***p < 0.001, ****p < 0.0001.

To determine whether Vpu can act as multifunctional protein capable of downregulating host proteins in the absence of neddylation and a functional SCF^β-TrCP^ complex, primary CD4^+^ T cells were infected with either an HIV-1_NL4-3_-derived, replication-defective virus carrying GFP in place of Nef (DHIVGFP(Vpu+/Nef−); Fig. 1a), or with an isogenic virus lacking both Nef and Vpu (DHIVGFP(Vpu−/Nef−); Fig. 1b) [21]. All viruses were pseudotyped with the vesicular stomatitis virus glycoprotein G (VSV-G). We utilized the above *nef*- and *env*-deficient viruses such that the known activities of Nef and Env on CD4 would not interfere with that of Vpu (reviewed in [22]). Two days post infection, cells were incubated in either DMSO (solvent) or MLN4924 and protein surface expression analyzed by flow cytometry 24 h later. As expected, the virus devoid of Nef and Vpu (DHIVGFP(Vpu−/Nef−)) showed similar surface levels of CD4, BST-2, CCR7 and NTB-A when comparing GFP-negative (uninfected) and -positive (infected) cells (Fig. 2a, panels ii, viii, xiv, xx). Downregulation of CD4, BST-2, CCR7 and NTB-A was apparent in cells that were infected with DHIVGFP(Vpu+/Nef−) and treated with DMSO (Fig. 2a, panels iii, vix, xv, xxi). However, MLN4924 relieved downmodulation of CD4 in a dose-dependent manner (Fig. 2a, panels iii–vi, b). In contrast, downregulation of BST-2, CCR7 and NTB-A was unaffected by MLN4924 treatment (Fig. 2a, panels vii–xxiv, b). These results indicate that Vpu utilizes both cullin-dependent and -independent mechanisms for down modulating host proteins.

### MLN4924 relieves NL4-3 Vpu, but not Nef mediated, degradation of CD4

**Fig. 3:**
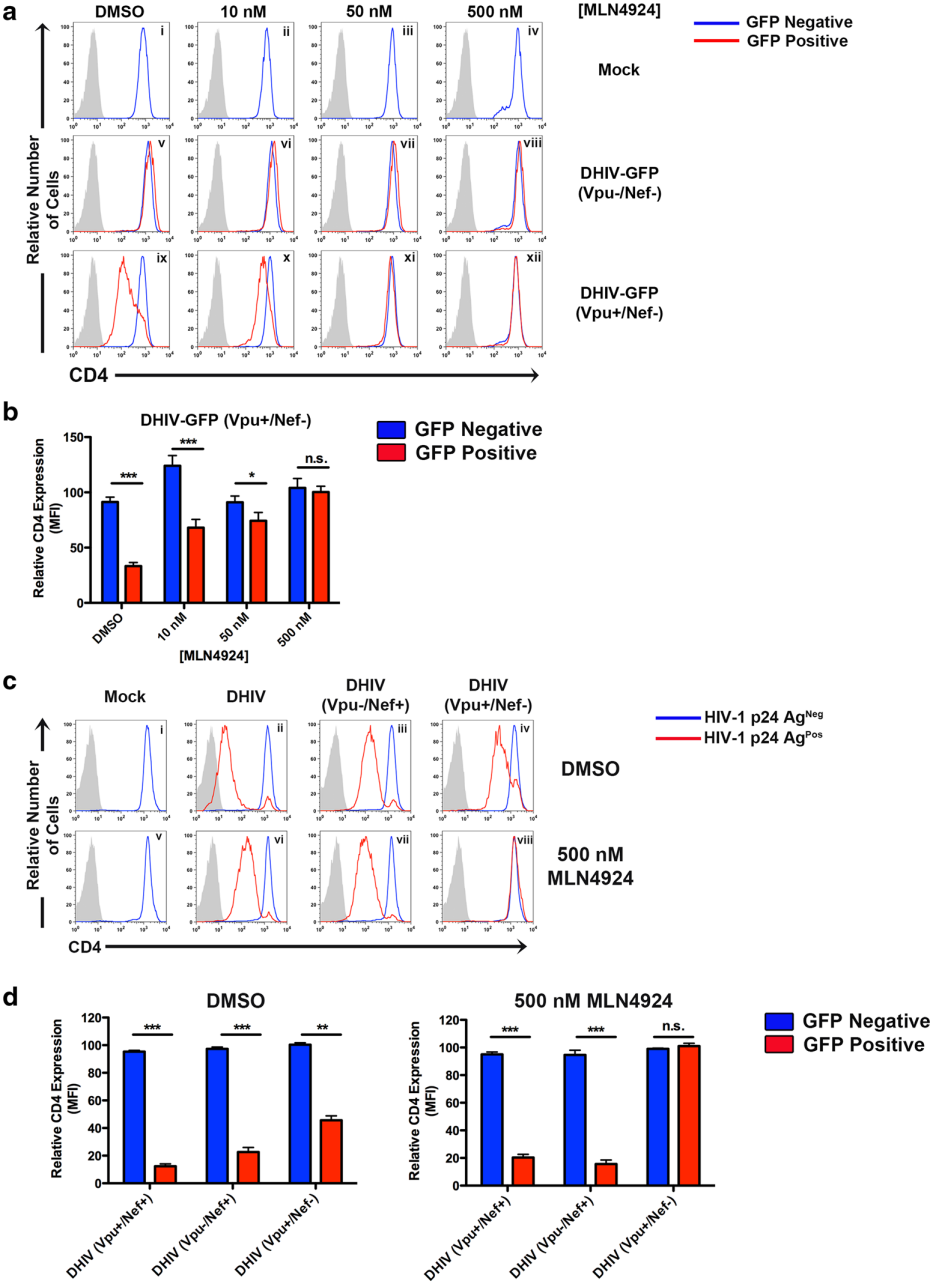
MLN4924 alleviates Vpu- but not Nef-mediated degradation of CD4. **a** Cultured T_CM_ were infected as described in Fig. 2a. To assess total levels of CD4, cells were permeabilized and stained 24 h after addition of MLN4924 and analyzed by flow cytometry. Histograms are color-coded as described in Fig. 2a. **b** MFI values of total (intracellular) CD4 expression levels from DHIVGFP(Vpu+Nef−). Data was normalized and statistical significance obtained as described in Fig. 2b. *n.s.* not significant. **c** Primary CD4^+^ T cells were either mock infected or infected at an MOI of 1 with DHIV WT, DHIV Vpu−/Nef+ or DHIV Vpu+/Nef−. 2 days post infection, cell cultures were treated with either DMSO or 500 nM MLN4924. 24 h post MLN4924 treatment, cells were assessed for surface levels of CD4 between p24Gag^neg^ (*blue line*) and p24Gag^pos^ (*red line*). *Gray* shaded histograms represent an IgG matched isotype control. Shown is one representative experiment out of three. **d** Relative CD4 surface levels were quantified from data obtained in Fig. 3c and are depicted graphically as ±SEM of either cells treated with DMSO (*left*) or 500 nM MLN4924 (*right*). Statistical significance between p24Gag^neg^ and p24Gag^pos^ populations was determined as in Fig. 2b.

To determine whether MLN4924 also prevented the degradation of CD4, primary CD4^+^ T cells were infected as described in Fig. 2 but were instead permeabilized, fixed and stained for total levels of CD4. Figure 3 shows that inhibition of neddylation rescued CD4 from Vpu-induced degradation (Fig. 3a, panels ix–xii, b). As a further control to show specificity of cullin inactivation by MLN4924, primary CD4^+^ T cells were infected with either env-defective HIV-1 (DHIV; Fig. 1e), lacking Vpu but expressing Nef (DHIV Vpu−/Nef+; Fig. 1f) or an isogenic virus lacking Nef and expressing Vpu (DHIV Vpu+/Nef−; Fig. 1g). Nef accelerates the endocytosis of target CD4 molecules present on the plasma membrane via clathrin and Adaptor-Protein 2 (AP-2), ultimately shuttling CD4 for lysosomal degradation in a multivesicular body (MVB) dependent manner [23, 24]. We therefore hypothesized that a virus encoding only Nef (DHIV Vpu−/Nef+) would be able to downmodulate CD4 in a manner that would be insensitive to MLN4924 treatment. This expectation was corroborated as shown in Figs. 3c (panels vii and viii), d.

### Primary Vpu isolates maintain the ability to downregulate BST-2 in the absence of CRL-activity

**Fig. 4:**
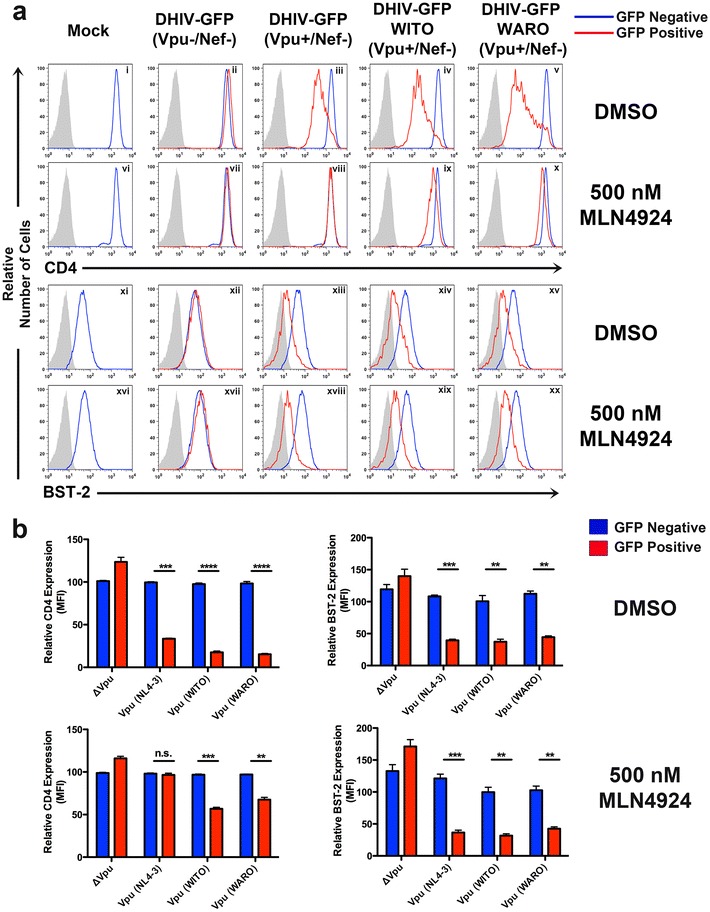
Primary Vpu isolates maintain the ability to decrease surface expression of CD4 and BST-2 in a CRL-dependent and independent manner. **a** Primary CD4^+^ T cells were either mock infected or infected at an MOI = 1 with DHIV-GFP (Nef−/Vpu−), DHIV-GFP (Nef−/Vpu+) or viruses encoding Vpu taken from either a transmitted/founder (T/F) (DHIV-GFP WITO (Nef−/Vpu+)) or a chronic carrier (CC) primary isolate (DHIV-GFP WARO (Nef−/Vpu+)). The cells were treated with either DMSO or 500 nM MLN4924 48 h post infection. 24 h post MLN treatment, the cellular GFP negative and positive populations (*red* and *blue* histograms) were assessed for surface levels of CD4 and BST-2 via flow cytometry. An IgG control was included to set the baseline for positive staining (*gray* shaded histogram). **b** Relative CD4 and BST-2 surface levels were quantified, normalized, and scored statistically as described in Fig. 2b.

A recent study showed that Vpu alleles from field strains of HIV-1 have the capacity to modulate host proteins, in particular CD4 and BST-2, to a greater extent than the prototypical HIV-1_NL4-3_ Vpu [25]. We therefore wished to determine whether the CRL-dependent and independent mechanisms observed with HIV-1_NL4-3_ Vpu (Fig. 2a) would be maintained with Vpu alleles from primary isolates. To address this, we replaced Vpu in DHIV-GFP(Vpu+/Nef−) with Vpu’s from either a transmitted founder (T/F; WITO) [26–30] or from a chronic carrier (CC; WARO) [31] virus. These new viruses were termed DHIV-GFP WITO (Vpu+/Nef−; Fig. 1c) and DHIV-GFP WARO (Vpu+/Nef−; Fig. 1d). We observed that Vpu’s from both a T/F (DHIV-GFP WITO) and CC (DHIV-GFP WARO) exhibited an enhanced ability to downregulate CD4 when compared to HIV-1_NL4-3_ Vpu (Fig. 4a, panels iii–v, b upper left). This effect was previously reported by Jafari et al., who proposed that primary Vpu isolates may adopt an optimized tertiary structure better suited to counteract CD4 as a result of differences between their amino acid sequence and that of HIV-1_NL4-3_ Vpu [16, 32, 33] (Fig. 1h). MLN4924 treatment had a dramatic effect on the ability of both a T/F and CC Vpu to downregulate CD4 (Fig. 4a, panels viii–x, b lower left). Both a T/F and CC Vpu were able to decrease the cell surface density of BST-2 to about the same extent as HIV-1_NL4-3_ Vpu (Fig. 4a, panels xiii–xv, b upper right). However, the addition of MLN4924 in cells expressing Vpu did not restore surface levels of BST-2 to any significant degree (Fig. 4a, panels xviii–xx, b lower right). These results reinforce the fact that primary Vpu isolates also possess the ability to counteract host proteins by CRL-independent mechanisms.

### siRNA mediated knockdown of cullin 1 reduces CD4, but not BST-2, surface levels in the presence of Vpu

**Fig. 5:**
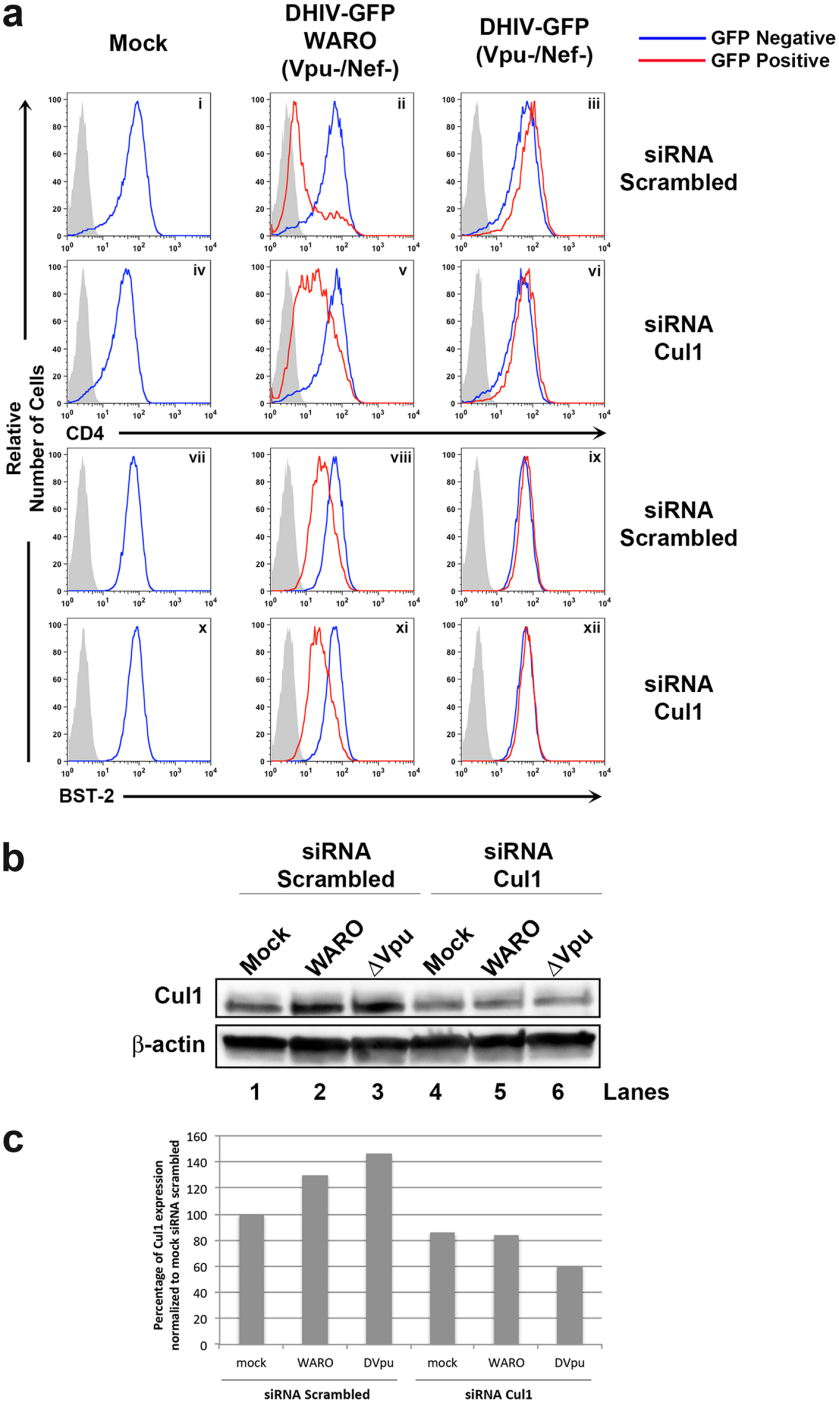
siRNA knockdown of cullin 1 hinders surface downmodulation of CD4, but not BST-2, by Vpu. **a** HeLa-CD4 cells were transfected twice with pooled control or cullin 1 siRNAs. 4 h post the second transfection, the cells were infected with VSV-G pseudotyped DHIV-GFP (Nef−/Vpu−) or DHIV-GFP WARO (Nef−/Vpu+). Cells were subsequently stained to detect surface levels of CD4 and BST-2 between GFP negative (*blue line*) and GFP positive (*red line*) populations 48 h post infection. *Gray* shaded histogram represents an IgG matched isotype control. **b** A portion of cells from **a** were lysed and subjected to Western blot to determine the knockdown efficiency of cullin 1. **c** Quantification of cullin 1 normalized to β-actin from **b**.

Although MLN4924 is primarily used and known as an NAE (and thus pan-CRL) inhibitor, at IC_50_ values of 1.5 and 8.2 μM the drug can also block the functions of the NAE-related enzymes, ubiquitin-activating enzyme (UAE) and SUMO-activating enzyme (SAE) [9]. Therefore, as an alternative approach to chemical inhibition, we assessed Vpu function in cells depleted of cullin 1. HeLa-CD4 cells were transfected twice (24 h apart) with either a non-targeting siRNA or siRNA targeting cullin 1, followed by infection with either DHIV-GFP WARO (Vpu+/Nef−) or DHIV-GFP (Vpu−/Nef−). As shown in Fig. 5, knockdown of cullin 1 attenuated Vpu’s capacity to downregulate CD4 from the cell surface (Fig. 5a, compare panels ii and v), but had no effect on downregulation of BST-2 (Fig. 5a, panels viii and xi). We conclude that Vpu’s ability to decrease surface levels of BST-2 is independent of CRL-activity.

## Discussion

Vpu acts as a functional protein that interferes with cellular targets through multiple mechanisms. The di-serine motif of Vpu renders Vpu capable of recruiting the E3 ubiquitin ligase complex substrate adaptor β-TrCP for target ubiquitination and eventual proteasomal (for CD4) [17, 34, 35] or endosomal sorting complexes required for transport (ESCRT) mediated endo-lysosomal degradation (for BST-2) [36–40]. Previous studies have also shown, however, that Vpu-mediated surface downregulation of BST-2 can be uncoupled from BST-2 degradation [41–43]. The explanation lies in the fact that Vpu induces the mislocalization of BST-2 within a perinuclear compartment (i.e. *trans*-golgi network (TGN)) [36, 41, 44–46]. As a consequence, both recycled and newly synthesized BST-2 are retained within the TGN, thereby decreasing total levels of BST-2 at the cell surface [45–47]. A recent report by Jia et al. denoted interaction of Vpu with the clathrin Adaptor-Protein complex 1 (AP-1) [19]. Binding of AP-1 and Vpu occurs through a conserved motif, E_59_xxxL_63_V_64_ (ELV), within Vpu’s C-terminal domain, previously reported to be important in BST-2 surface downmodulation and viral release [18]. In our present study, we found that pharmacological inhibition of CRL- activity or knock down of cullin 1 hindered Vpu’s capacity to downregulate CD4, but not BST-2. Therefore, our findings support a model whereby cullin activity (and β-TrCP) are dispensable for Vpu to downregulate BST-2.

Numerous studies have shown that the di-serine motif of Vpu, which mediates interaction with β-TrCP when phosphorylated, is required for downmodulation and/or degradation of both CD4 and BST-2 [17, 48–52]. Thus, mutation of the di-serine motif abrogates degradation of CD4 [16, 53], confirming a role for β-TrCP. On the other hand, Tervo et al. found that depletion of β-TrCP2 or the simultaneous depletion of β-TrCP1 and 2 did not block the ability of Vpu to promote HIV-1 release and failed to restore surface levels of BST-2 [43]. These results, taken together, suggest that the di-serine motif of Vpu is directly involved in interaction with β-TrCP, but that mutations in this motif affect the downregulation of BST-2 through possibly a more general effect on Vpu protein conformation or perhaps the binding of another cellular factor implicated in the mislocalization of host proteins [43]. Therefore, our observation that downregulation of BST-2, CCR7 and NTB-A is CRL-independent does not contradict the notion that the di-serine motif of Vpu is required for this function [15, 21, 54].

Structurally, phosphorylation of Vpu at serine 52 and 56 induces a conformational change in Vpu’s C-terminus: the formation of a β-strand within residues 50–59 and displacement of the third alpha helix (h3) away from the phosphorylation site [55]. The structural rearrangements induced by phosphorylation result in the emergence of acidic side chains surrounding serine 52 and 56, creating an electronegative binding region upon which a protein exhibiting electropositive potential can bind [55]. In particular, phosphorylated serines 52 and 56, glycine 53, asparagine 54 and the hydrophobic residues isoleucine 46 and leucine 45 were shown to be involved in the binding to the F-box protein β-TrCP [56]. Our observations with MLN4924 and cullin knockdown argue against a role for cullin mediated ubiquitination in the downregulation of BST-2 from an independent line of experimentation other than mutagenesis.

Given that Vpu does not induce the degradation of CCR7 or NTB-A but rather retention within the TGN, our data suggest that these molecules are also downregulated in a CRL-independent manner (Fig. 2a) [20, 21, 54]. A recent report by Bachle et al. identified a conserved C-terminal AWF motif present within HIV-1 subtype B Vpu isolates that influences the ability of Vpu to downregulate the lipid antigen receptor CD1d [57]. Therefore, whether Vpu’s AWF or ELV motifs, which are CRL-independent, are required for downmodulation of CCR7 and NTB-A remains to be determined. It will also be compelling to investigate the requirement for β-TrCP and the SCF^β-TrCP^ complex in downmodulation of other reported targets of Vpu, such as CD1d, CD155, CD62L and members of the tetraspanin family [58–61].

## Conclusions

This work shows that cullin inactivation, through either pharmacological inhibition or depletion of cullin 1, does not render Vpu unable to downregulate BST-2, CCR7 or NTB-A. This highlights that facts that Vpu is multifunctional and that therapeutic targeting of neddylation, while potent and specific against CD4 downmodulation by Vpu, would still allow other targets to be downregulated.

## Methods

### Antibodies and reagents

Antibodies used in this study were as follows: APC-labeled mouse anti-human CCR7 (clone 150303; R & D Systems Inc.), mouse anti-human NTB-A-APC (clone 292811; R & D Systems Inc.), APC-labeled mouse anti-human CD4 (clone S3.5; Life Technologies), rabbit anti-human BST-2 (NIH AIDS Reagent Program, Division of AIDS, NIAID, NIH; Drs. Klaus Strebel and Amy Andrew (Cat. # 11721)), goat-anti-rabbit Alexa Fluor (AF) 647 (Molecular Probes, Invitrogen), mouse APC-conjugated isotype control (clone 20102; R & D Systems Inc.), rabbit AF-647-conjugated isotype control (Cell Signaling Technology), rabbit anti-human cullin 1 (abcam) and mouse anti-human β-actin (Sigma Aldrich). MLN4924 was purchased from Cayman Biologicals. The dry solvent was then resuspended in DMSO at a stock concentration of 20 mM, further aliquotted and diluted at 200 μM and used as indicated. ON-TARGETplus SMARTpool siRNAs targeting human cullin 1 or control non-targeting siRNAs were purchased from Dharmacon.

### Cells and plasmids

Human embryonic kidney 293T (HEK293T) cells were maintained in Dulbecco’s Modified Eagle’s Medium (DMEM) containing 10% fetal bovine serum (Atlas Biologicals) supplemented with 100 U/ml penicillin, 100 μg/ml streptomycin and 2 mM l-glutamine (Life Technologies). HeLa-CD4^+^ clone 1022 (obtained through the NIH AIDS Reagent Division of AIDS, NIAID, NIH (Cat. #1109; Dr. Bruce Chesebro) were cultured in RPMI complete media in the presence of 1 μg/ml G418 (Life Technologies) while CCRF-CEM and primary CD4^+^ T cells were cultured in RPMI complete media only. All cells were maintained at 5% CO_2_ at 37°C. For all experiments involving primary CD4^+^ T cells, coverage was maintained under protocol #IRB_00067637 approved by the University of Utah Institutional Review Board. The generation of cultured T_CM_ has been described previously [21].

The DHIV plasmids used in this manuscript have been described previously (Ramirez et al.; Cell Reports 2014). To construct DHIV-GFP WITO (Vpu+/Nef−) and DHIV-GFP WARO (Vpu+/Nef−), we first re-ligated DHIV-GFP (Vpu+/Nef−) after Xho1 and Sma1 digestion to create a unique EcoR1 site. A novel Mlu1 site after Vpu (ACC TGT to ACGCGT) was then introduced using site-directed mutagenesis (Stratagene) according to the manufacturers’ instructions with the following primers: FWD 5′-CAG TCTATTATGGGGTACGCGTGTGGAAGGAAGCAACC and REV 5′-GGTTGCTTC CTTCCACACGCGTACCCCATAATAGACTG. To replace HIV-1_NL4-3_ Vpu with Vpu’s from primary isolates, we obtained full length transmitted founder (T/F; pWITO.c/2474; Cat# 11739, Dr. John Kappes and Dr. Christina Ochsenbauer) or chronic carrier (CC; pWARO; Cat # 12419, Dr. Beatrice Hahn) HIV-1 infectious molecular clones (IMC) from the NIH AIDS Reagent Program. HIV-1_WITO_ Vpu was PCR amplified using the following primers: FWD 5′-GCAGGAGTGGAAGCCATAATAA*GAATTC* and REV 5′-ACA*ACGCGT*CTACTCATCATTAACATCCCAAGGAGC (EcoR1 and Mlu1 sites italicized, respectively) and subcloned into DHIV-GFP(Vpu+/Nef−) to create DHIV-GFP WITO(Vpu+/Nef−). DHIV-GFP WARO(Vpu+/Nef−) was constructed in a similar fashion with the following HIV-1_WARO_ Vpu primers: FWD 5′-GGAGTGGAAGCCATAATAA*GAATTC*TGC and REV 5′-ACG*ACGCGT*CTACAGATCATTAATATCCCAAGGAGCATC. All constructs were confirmed via sequencing.

### Flow cytometry

Surface levels of CD4, BST-2, CCR7 and NTB-A were assessed by staining cells with their appropriate antibodies at 4°C for 30 min in buffer (1 × PBS + 3% FBS). An additional step including a secondary antibody was necessary to detect BST-2 surface levels. A viability dye, eFlour 450 (eBioscience) was then used to distinguish live from dead cells. Fixation was achieved using 0.5% Paraformaldehyde (PFA).

In experiments involving surface analysis of CD4 and detection of intracellular p24, cells were first probed with anti-APC-CD4, stained with eFluor 450, permeabilized (Cytofix/Cytoperm: BD Biosciences) and then stained with mouse-anti-FITC-p24. Total levels of CD4 in primary CD4^+^ T cells were measured by staining cells with eFlour 450, permeabilization and then probing with anti-APC-CD4. All data was collected on a BD FACS CantoII and analyzed with FlowJo software.

### Viruses and infections

Viral stocks were generated by co-transfection of 20 μg DHIV along with 5 μg of a construct expressing vesicular stomatitis virus G-protein (VSV-G) by calcium phosphate mediated transfection into HEK293T cells. Media (DMEM) was replaced after 16 h and the cellular supernatant collected, aliquotted and stored at −80°C 48 h post-transfection. Viral titers and MOI were determined via infection of CCRF-CEM cells. Primary CD4^+^ T cells generated as described above were infected 5 days post-activation at an MOI of 1 via spinoculation: 10^6^ cells (1 ml final volume) for 2 h at 37°C in the presence of 8 μg/ml Polybrene (Sigma). After infection, cells were then resuspended in RPMI complete medium supplemented with IL-2 (30 U/ml).

### siRNA mediated cullin 1 depletion

HeLa cells (5 × 10^5^) were transfected twice (24 h apart) with either control siRNA or siRNAs against cullin 1 at a final concentration of 100 nM using Lipofectamine 2000 (Invitrogen) according to the manufacturers’ instructions. The medium was changed 4 h after each transfection. 4 h post the second transfection, the cells were either mock infected or infected with DHIV-GFP (Vpu−/Nef−) or DHIV-GFP WARO (Vpu+/Nef−) for 4 h at 37°C at an MOI = 0.8. After 48 h, the cells were either processed for flow cytometry or lysed and subjected to Western blot to detect cellular levels of cullin 1 between samples.

## Abbreviations

CRL: cullin-RING ligase
RING: really interesting new gene
AP-1: Adaptor-Protein 1
Vpu: viral protein u

## References

[1] Petroski MD, Deshaies RJ (2005). Function and regulation of cullin-RING ubiquitin ligases. Nat Rev Mol Cell Biol.

[2] Harris RS, Bishop KN, Sheehy AM, Craig HM, Petersen-Mahrt SK, Watt IN (2003). DNA deamination mediates innate immunity to retroviral infection. Cell.

[3] Marin M, Rose KM, Kozak SL, Kabat D (2003). HIV-1 Vif protein binds the editing enzyme APOBEC3G and induces its degradation. Nat Med.

[4] Stopak K, de Noronha C, Yonemoto W, Greene WC (2003). HIV-1 Vif blocks the antiviral activity of APOBEC3G by impairing both its translation and intracellular stability. Mol Cell.

[5] Yu X, Yu Y, Liu B, Luo K, Kong W, Mao P (2003). Induction of APOBEC3G ubiquitination and degradation by an HIV-1 Vif-Cul5-SCF complex. Science.

[6] Hrecka K, Hao C, Gierszewska M, Swanson SK, Kesik-Brodacka M, Srivastava S (2011). Vpx relieves inhibition of HIV-1 infection of macrophages mediated by the SAMHD1 protein. Nature.

[7] Laguette N, Sobhian B, Casartelli N, Ringeard M, Chable-Bessia C, Ségéral E (2011). SAMHD1 is the dendritic- and myeloid-cell-specific HIV-1 restriction factor counteracted by Vpx. Nature.

[8] Bosu DR, Kipreos ET (2008). Cullin-RING ubiquitin ligases: global regulation and activation cycles. Cell Div.

[9] Soucy TA, Smith PG, Milhollen MA, Berger AJ, Gavin JM, Adhikari S (2009). An inhibitor of NEDD8-activating enzyme as a new approach to treat cancer. Nature.

[10] Stanley DJ, Bartholomeeusen K, Crosby DC, Kim DY, Kwon E, Yen L (2012). Inhibition of a NEDD8 cascade restores restriction of HIV by APOBEC3G. PLoS Pathog.

[11] Hofmann H, Norton TD, Schultz ML, Polsky SB, Sunseri N, Landau NR (2013). Inhibition of CUL4A neddylation causes a reversible block to SAMHD1-mediated restriction of HIV-1. J Virol.

[12] Nekorchuk MD, Sharifi HJ, Furuya AKM, Jellinger R, de Noronha CMC (2013). HIV relies on neddylation for ubiquitin ligase-mediated functions. Retrovirology.

[13] Wei W, Guo H, Liu X, Zhang H, Qian L, Luo K (2014). A first-in-class NAE inhibitor, MLN4924, blocks lentiviral infection in myeloid cells by disrupting neddylation-dependent Vpx-mediated SAMHD1 degradation. J Virol.

[14] Neil SJD, Zang T, Bieniasz PD (2008). Tetherin inhibits retrovirus release and is antagonized by HIV-1 Vpu. Nature.

[15] Van Damme N, Goff D, Katsura C, Jorgenson RL, Mitchell R, Johnson MC (2008). The interferon-induced protein BST-2 restricts HIV-1 release and is downregulated from the cell surface by the viral Vpu protein. Cell Host Microbe.

[16] Schubert U, Strebel K (1994). Differential activities of the human immunodeficiency virus type 1-encoded Vpu protein are regulated by phosphorylation and occur in different cellular compartments. J Virol.

[17] Margottin F, Bour SP, Durand H, Selig L, Benichou S, Richard V (1998). A novel human WD protein, h-beta TrCp, that interacts with HIV-1 Vpu connects CD4 to the ER degradation pathway through an F-box motif. Mol Cell.

[18] Kueck T, Neil SJD (2012). A cytoplasmic tail determinant in HIV-1 Vpu mediates Targeting of tetherin for endosomal degradation and counteracts interferon-induced restriction. PLoS Pathog.

[19] Jia X, Weber E, Tokarev A, Lewinski M, Rizk M, Suarez M et al (2014) Structural basis of HIV-1 Vpu-mediated BST2 antagonism via hijacking of the clathrin adaptor protein complex 1. eLife 3:e02362 10.7554/eLife.02362 PMC4018625 24843023

[20] Shah AH, Sowrirajan B, Davis ZB, Ward JP, Campbell EM, Planelles V (2010). Degranulation of natural killer cells following interaction with HIV-1-infected cells is hindered by downmodulation of NTB-A by Vpu. Cell Host Microbe.

[21] Ramirez PW, Famiglietti M, Sowrirajan B, DePaula-Silva AB, Rodesch C, Barker E (2014). Downmodulation of CCR7 by HIV-1 Vpu results in impaired migration and chemotactic signaling within CD4(+) T cells. Cell Rep.

[22] Malim MH, Emerman M (2008). HIV-1 accessory proteins—ensuring viral survival in a hostile environment. Cell Host Microbe.

[23] Chaudhuri R, Lindwasser OW, Smith WJ, Hurley JH, Bonifacino JS (2007). Downregulation of CD4 by human immunodeficiency virus type 1 Nef is dependent on clathrin and involves direct interaction of Nef with the AP2 clathrin adaptor. J Virol.

[24] daSilva LLP, Sougrat R, Burgos PV, Janvier K, Mattera R, Bonifacino JS (2009). Human immunodeficiency virus type 1 Nef protein targets CD4 to the multivesicular body pathway. J Virol.

[25] Pickering S, Hué S, Kim E-Y, Reddy S, Wolinsky SM, Neil SJD (2014). Preservation of tetherin and CD4 counter-activities in circulating Vpu alleles despite extensive sequence variation within HIV-1 infected individuals. PLoS Pathog.

[26] Keele BF, Giorgi EE, Salazar-Gonzalez JF, Decker JM, Pham KT, Salazar MG (2008). Identification and characterization of transmitted and early founder virus envelopes in primary HIV-1 infection. Proc Natl Acad Sci USA.

[27] Salazar-Gonzalez JF, Bailes E, Pham KT, Salazar MG, Guffey MB, Keele BF (2008). Deciphering human immunodeficiency virus type 1 transmission and early envelope diversification by single-genome amplification and sequencing. J Virol.

[28] Lee HY, Giorgi EE, Keele BF, Gaschen B, Athreya GS, Salazar-Gonzalez JF (2009). Modeling sequence evolution in acute HIV-1 infection. J Theor Biol.

[29] Salazar-Gonzalez JF, Salazar MG, Keele BF, Learn GH, Giorgi EE, Li H (2009). Genetic identity, biological phenotype, and evolutionary pathways of transmitted/founder viruses in acute and early HIV-1 infection. J Exp Med.

[30] Ochsenbauer C, Edmonds TG, Ding H, Keele BF, Decker J, Salazar MG (2012). Generation of transmitted/founder HIV-1 infectious molecular clones and characterization of their replication capacity in CD4 T lymphocytes and monocyte-derived macrophages. J Virol.

[31] Parrish NF, Gao F, Li H, Giorgi EE, Barbian HJ, Parrish EH (2013). Phenotypic properties of transmitted founder HIV-1. Proc Natl Acad Sci USA.

[32] Magadan JG, Bonifacino JS (2012). Transmembrane domain determinants of CD4 downregulation by HIV-1 Vpu. J Virol.

[33] Jafari M, Guatelli J, Lewinski MK (2014). Activities of transmitted/founder and chronic clade B HIV-1 Vpu and a C-terminal polymorphism specifically affecting virion release. J Virol.

[34] Willey RL, Maldarelli F, Martin MA, Strebel K (1992). Human immunodeficiency virus type 1 Vpu protein induces rapid degradation of CD4. J Virol.

[35] Magadan JG, Perez-Victoria FJ, Sougrat R, Ye Y, Strebel K, Bonifacino JS (2010). Multilayered mechanism of CD4 downregulation by HIV-1 Vpu involving distinct ER retention and ERAD targeting steps. PLoS Pathog.

[36] Douglas JL, Viswanathan K, McCarroll MN, Gustin JK, Früh K, Moses AV (2009). Vpu directs the degradation of the human immunodeficiency virus restriction factor BST-2/tetherin via a {beta}TrCP-dependent mechanism. J Virol.

[37] Iwabu Y, Fujita H, Kinomoto M, Kaneko K, Ishizaka Y, Tanaka Y (2009). HIV-1 accessory protein Vpu internalizes cell-surface BST-2/tetherin through transmembrane interactions leading to lysosomes. J Biol Chem.

[38] Mitchell RS, Katsura C, Skasko MA, Fitzpatrick K, Lau D, Ruiz A (2009). Vpu antagonizes BST-2-mediated restriction of HIV-1 release via beta-TrCP and endo-lysosomal trafficking. PLoS Pathog.

[39] Caillet M, Janvier K, Pelchen-Matthews A, Delcroix-Genête D, Camus G, Marsh M (2011). Rab7A is required for efficient production of infectious HIV-1. PLoS Pathog.

[40] Janvier K, Pelchen-Matthews A, Renaud J-B, Caillet M, Marsh M, Berlioz-Torrent C (2011). The ESCRT-0 component HRS is required for HIV-1 Vpu-mediated BST-2/tetherin down-regulation. PLoS Pathog.

[41] Dubé M, Roy BB, Guiot-Guillain P, Binette J, Mercier J, Chiasson A (2010). Antagonism of tetherin restriction of HIV-1 release by Vpu involves binding and sequestration of the restriction factor in a perinuclear compartment. PLoS Pathog.

[42] Goffinet C, Homann S, Ambiel I, Tibroni N, Rupp D, Keppler OT (2010). Antagonism of CD317 restriction of human immunodeficiency virus type 1 (HIV-1) particle release and depletion of CD317 are separable activities of HIV-1 Vpu. J Virol.

[43] Tervo HM, Homann S, Ambiel I, Fritz JV, Fackler OT, Keppler OT (2011). beta-TrCP is dispensable for Vpu’s ability to overcome the CD317/tetherin-imposed restriction to HIV-1 release. Retrovirology.

[44] Hauser H, Lopez LA, Yang SJ, Oldenburg JE, Exline CM, Guatelli JC (2010). HIV-1 Vpu and HIV-2 Env counteract BST-2/tetherin by sequestration in a perinuclear compartment. Retrovirology.

[45] Lau D, Kwan W, Guatelli J (2011). Role of the endocytic pathway in the counteraction of BST-2 by human lentiviral pathogens. J Virol.

[46] Schmidt S, Fritz JV, Bitzegeio J, Fackler OT, Keppler OT (2011) HIV-1 Vpu blocks recycling and biosynthetic transport of the intrinsic immunity factor CD317/tetherin to overcome the virion release restriction. MBio 2(3):e00036–11. doi:10.1128/mBio.00036-11 10.1128/mBio.00036-11 PMC3101777 21610122

[47] Dube M, Paquay C, Roy BB, Bego MG, Mercier J, Cohen EA (2011). HIV-1 Vpu antagonizes BST-2 by interfering mainly with the trafficking of newly synthesized BST-2 to the cell surface. Traffic.

[48] Chen MY, Maldarelli F, Karczewski MK, Willey RL, Strebel K (1993). Human immunodeficiency virus type 1 Vpu protein induces degradation of CD4 in vitro: the cytoplasmic domain of CD4 contributes to Vpu sensitivity. J Virol.

[49] Schubert U, Bour S, Ferrer-Montiel AV, Montal M, Maldarell F, Strebel K (1996). The two biological activities of human immunodeficiency virus type 1 Vpu protein involve two separable structural domains. J Virol.

[50] Goffinet C, Allespach I, Homann S, Tervo HM, Habermann A, Rupp D (2009). HIV-1 antagonism of CD317 is species specific and involves Vpu-mediated proteasomal degradation of the restriction factor. Cell Host Microbe.

[51] Mangeat B, Gers-Huber G, Lehmann M, Zufferey M, Luban J, Piguet V (2009). HIV-1 Vpu neutralizes the antiviral factor tetherin/BST-2 by binding it and directing its beta-TrCP2-dependent degradation. PLoS Pathog.

[52] Schindler M, Rajan D, Banning C, Wimmer P, Koppensteiner H, Iwanski A (2010). Vpu serine 52 dependent counteraction of tetherin is required for HIV-1 replication in macrophages, but not in ex vivo human lymphoid tissue. Retrovirology.

[53] Margottin F, Benichou S, Durand H, Richard V, Liu LX, Gomas E (1996). Interaction between the cytoplasmic domains of HIV-1 Vpu and CD4: role of Vpu residues involved in CD4 interaction and in vitro CD4 degradation. Virology.

[54] Bolduan S, Hubel P, Reif T, Lodermeyer V, Höhne K, Fritz JV (2013). HIV-1 Vpu affects the anterograde transport and the glycosylation pattern of NTB-A. Virology.

[55] Coadou G, Evrard-Todeschi N, Gharbi-Benarous J, Benarous R, Girault JP (2002). HIV-1 encoded virus protein U (Vpu) solution structure of the 41-62 hydrophilic region containing the phosphorylated sites Ser52 and Ser56. Int J Biol Macromol.

[56] Coadou G, Gharbi-Benarous J, Megy S, Bertho G, Evrard-Todeschi N, Segeral E (2003). NMR studies of the phosphorylation motif of the HIV-1 protein Vpu bound to the F-box protein beta-TrCP. Biochemistry.

[57] Bachle SM, Sauter D, Sibitz S, Sandberg JK, Kirchhoff F, Moll M (2015). Involvement of a C-terminal motif in the interference of primate lentiviral Vpu proteins with CD1d-mediated antigen presentation. Sci Rep.

[58] Moll M, Andersson SK, Smed-Sörensen A, Sandberg JK (2010). Inhibition of lipid antigen presentation in dendritic cells by HIV-1 Vpu interference with CD1d recycling from endosomal compartments. Blood.

[59] Matusali G, Potesta M, Santoni A, Cerboni C, Doria M (2012). The human immunodeficiency virus type 1 Nef and Vpu proteins downregulate the natural killer cell-activating ligand PVR. J Virol.

[60] Haller C, Müller B, Fritz JV, Lamas-Murua M, Stolp B, Pujol F (2014). HIV-1 Nef and Vpu are functionally redundant broad-spectrum modulators of cell surface receptors including tetraspanins. J Virol.

[61] Vassena L, Giuliani E, Koppensteiner H, Bolduan S, Schindler M, Doria M (2015). HIV-1 Nef and Vpu interfere with L-selectin (CD62L) cell surface expression to inhibit adhesion and signaling in infected CD4+ T lymphocytes. J Virol.

